# ZnO Structures with Surface Nanoscale Interfaces Formed by Au, Fe_2_O_3_, or Cu_2_O Modifier Nanoparticles: Characterization and Gas Sensing Properties

**DOI:** 10.3390/s21134509

**Published:** 2021-06-30

**Authors:** Milena Tomić, Martha Claros, Isabel Gràcia, Eduard Figueras, Carles Cané, Stella Vallejos

**Affiliations:** 1 Institute of Microelectronics of Barcelona (IMB-CNM, CSIC), Campus UAB, Cerdanyola del Vallès, 08193 Barcelona, Spain; milena.tomic@imb-cnm.csic.es (M.T.); isabel.gracia@imb-cnm.csic.es (I.G.); eduard.figueras@imb-cnm.csic.es (E.F.); carles.cane@imb-cnm.csic.es (C.C.); 2 Department of Electronic Engineering, Campus UAB, Autonomous University of Barcelona (UAB), Cerdanyola del Vallès, 08193 Barcelona, Spain; 3 CEITEC-Central European Institute of Technology, Brno University of Technology, 61200 Brno, Czech Republic; martha.claros@ceitec.vutbr.cz

**Keywords:** zinc oxide, gold, iron oxide, copper oxide, interfaces, Schottky junctions, heterojunctions, gas sensing, nitrogen dioxide

## Abstract

Zinc oxide rod structures are synthetized and subsequently modified with Au, Fe_2_O_3_, or Cu_2_O to form nanoscale interfaces at the rod surface. X-ray photoelectron spectroscopy corroborates the presence of Fe in the form of oxide—Fe_2_O_3_; Cu in the form of two oxides—CuO and Cu_2_O, with the major presence of Cu_2_O; and Au in three oxidation states—Au^3+^, Au^+^, and Au^0^, with the content of metallic Au being the highest among the other states. These structures are tested towards nitrogen dioxide, ethanol, acetone, carbon monoxide, and toluene, finding a remarkable increase in the response and sensitivity of the Au-modified ZnO films, especially towards nitrogen dioxide and ethanol. The results for the Au-modified ZnO films report about 47 times higher response to 10 ppm of nitrogen dioxide as compared to the non-modified structures with a sensitivity of 39.96% ppm^−1^ and a limit of detection of 26 ppb to this gas. These results are attributed to the cumulative effects of several factors, such as the presence of oxygen vacancies, the gas-sensing mechanism influenced by the nano-interfaces formed between ZnO and Au, and the catalytic nature of the Au nanoparticles.

## 1. Introduction

Gas sensors are applied in diverse scenarios, including industry, environmental protection, medicine, food quality, public safety, etc., and their usage is projected to increase further as the Internet of Things (IoT) settles [1]. Amongst a host of available gas sensor technologies based on nanomaterials, metal oxide semiconductors (MOX) based gas sensors are very promising due to their good sensitivity, small dimensions, compatibility with microelectronic fabrication processes, and potentially low cost. These devices have shown sensitivity to various gases (e.g., CO, NO_2_, NH_3_, H_2_S) and vapors (alcohols, ketones, aldehydes, aromatic compounds), although their performance in terms of limits of detection and selectivity is still a subject of improvement [2,3,4].

Since the gas sensing response and mechanism of MOX are surface-dependent, previous research on gas-sensitive MOXs and their functional properties (e.g., sensitivity, selectivity, stability, speed of response) found it significant to reduce the particle size of these materials to improve their properties [5]. Other studies, moreover, showed that crystalline structures with specific surface morphologies (e.g., rods, wires, plates, etc.) are determinants to achieve better gas sensing properties [6]. The modification of these nanosized crystalline structures with second-phase constituents, either noble metals or other MOXs, demonstrated another via to enhance the functionality of the MOX support to different gases and vapors. These improvements are generally connected with the increment of adsorption centers at the surface and the band energy structure modification due to the formation of Schottky-barrier-like junctions (metal–MOX) or heterojunctions (MOX–MOX) [7]. Consequently, the interest in finding synthetic methods that allow for crystalline MOXs with tuned second-phase constituents (metals or MOXs) has kept constant in the gas sensing field, as well as the use of methods that provide scalability to these materials and compatibility with state-of-the-art microfabrication processes for possible multi-purpose microelectronic devices [3,7].

Zinc oxide (ZnO) is an n-type semiconductor with a wide band-gap (E_G_ = 3.3 eV), high chemical and thermal stability, and tunable surface morphology due to its stable wurtzite structure [8,9]. This MOX, in its pristine form and modified with noble metals (e.g., Au, Ag, Pt, or Pd) [10,11,12,13] or another MOX (e.g., CuO) [14], is one of the most representative gas-sensitive MOXs with proved sensitivity to gases, such as CO, H_2_, H_2_S, and NO_2_, and vapors such as ethanol, methanol, and acetaldehyde [3]. ZnO was synthesized previously by different methods, including wet- and vapor-chemical routes, delivering films and powders with varied forms, e.g., nanoplates, nanospheres, nanowires, nanorods, nanotubes, nanoflowers, nanofibers, nanoneedles, and nanoribbons [3,4]. Recently, we developed a method to deposit ZnO by aerosol-assisted chemical vapor deposition (AACVD), which offers significant advantages over other CVD techniques, including atmospheric pressure and relatively low-temperature process, as well as the possibility of tuning the material morphology and structure [15,16]. This method allowed the formation of crystalline rod-like ZnO structures and their direct integration into microsensors [17]. The surface modification of MOX structures with second-phase constituents can also be achieved by different methods [7]; amongst them, AACVD [18,19,20] and impregnation [21,22] methods proved effective to modify structures with both metals and MOXs. Recently, for instance, AACVD was employed to modify ZnO with iron and copper [3], although the gas sensing performance of these systems was not evaluated. Similarly, impregnation procedures based on the reduction of a chemical inorganic precursor were used to incorporate Ag and Au particles over ZnO [23,24]. However, these procedures usually do not include gold and silver preformed nanoparticles, as in the present work, and they were not employed for modifying gas-sensitive ZnO structures.

Here, we use these synthetic methods to develop crystalline ZnO structures modified with Au, Cu_2_O, and Fe_2_O_3_ particles and go further by exploring their applicability on gas sensing. The work includes a broad analysis of the properties of these materials with nanoscale interfaces towards several gases, such as nitrogen dioxide, ethanol, acetone, carbon monoxide, and toluene, and a discussion of their influence on the gas sensing mechanism.

## 2. Materials and Methods

ZnO rod structures were synthesized at 400 °C on Si/SiO_2_ tiles (40 mm × 40 mm) and Si-based transducing platforms (40 mm × 40 mm) via the AACVD of ZnCl_2_ (50 mg, ≥98.0%, Sigma–Aldrich, St. Louis, MO, USA) dissolved in ethanol (5 mL, ≥99.9%, PanReac, Chicago, IL, USA), as described earlier [17]. The transducing platforms contained 100 pairs of Cr/Au (40/200 nm thick) interdigitated electrodes (5 × 2000 μm) with an electrode gap of 5 μm isolated from the Si substrate by a SiO_2_ layer. The ZnO structures were modified with Au, Ag, Fe_2_O_3_, and Cu_2_O in a second step using AACVD or impregnation with preformed colloidal nanoparticles. The AACVD of FeCl_3_·6H_2_O (3 mg, ≥99.0%, Sigma–Aldrich) at 430 °C and Cu(NO_3_)_2_·6H_2_O (3 mg, ≥99.9%, Sigma–Aldrich) at 450 °C dissolved in acetone (3 mL, ≥99.9%, PanReac) and ethanol (3 mL, ≥99.9%, PanReac), respectively, allowed the incorporation of iron and copper oxides at the ZnO rods, similarly to our previous studies [25]. However, the AACVD at 400 °C of ethanolic solutions (3 mL) of HAuCl_4_·3H_2_O or AgNO_3_ in concentrations up to 19 mM did not show appropriate for the incorporation of Au and Ag at the ZnO rods, despite previous evidence of the AACVD of these solutions on Si and glass tiles and other materials including WO_3_ structures [26,27]. Hence, the ZnO structures were modified with Au and Ag by impregnation method using (preformed) colloidal nanoparticles synthesized by chemical reduction of HAuCl_4_·3H_2_O (≥99.9%, Sigma–Aldrich) or AgNO_3_ (≥99.0%, Sigma–Aldrich) with Na_3_C_6_H_5_O_7_·2H_2_O (Ph Eur, BP, JP, USP, E 331, Sigma–Aldrich) [28] or NaBH_4_ (99.0%, Sigma–Aldrich) [29], respectively. The as-synthesized nanoparticles were washed with distilled water by centrifugation (12,000 rpm, 30 min) to eliminate the unreacted chemicals before the impregnation process, which consisted of immersing the ZnO samples (60 s) into the solution with suspended preformed Au or Ag colloids heated at 60 °C.

The samples were annealed in synthetic air at 450 °C for 1 h. An examination of the samples’ morphology before and after annealing using SEM (Scanning Electron Microscopy—Zeiss Auriga series, 3 kV) did not show noticeable changes. The samples synthesized on Si tiles were used to perform most of the physical and chemical analysis to avoid possible interferences from the Cr/Au electrodes, whereas the Si-based transducing platforms were used primarily for gas sensing tests after performing SEM and confirm the integration of structures with similar characteristics to those registered on the Si tiles. An analysis of the samples synthesized on Si-tiles involved the examination of morphology by TEM (Transmission Electron Microscopy—FEI Tecnai, 200 kV), including STEM (Scanning Transmission Electron Microscopy) mode with elemental analysis by EDS (Energy Dispersive Spectroscopy) and HRTEM (High-Resolution Transmission Electron Microscopy) mode. The crystalline structure was analyzed by XRD (X-ray Diffraction—Bruker, AXS D8 Advance, Cu Kα radiation operated at 40 kV and 40 mA), and the chemical composition by XPS (X-ray Photoelectron Spectroscopy—Kratos Axis Supra spectrometer, with Al/Ag monochromatic X-ray source).

The structures were tested in a continuous flow system with dry air as the reference gas and ethanol, acetone, toluene, carbon monoxide, and nitrogen dioxide either in dry or humid ambient as analyte gases. After the exposure (10 min) to each gas analyte, the structures were recovered in the air for 30 min. Humidity was introduced to the system by bubbling through the water under controlled conditions at 25 °C. The relative humidity (RH) just at the outlet of the test chamber was monitored throughout the whole test using a humidity/temperature sensor (SHT71, operating ranges from 0 to 100% RH, accuracy of ±3% RH and ±0.4 °C) [25]. The sensor response (R) was defined as (Ra-Rg)/Ra for the reducing gases and (Rg-Ra)/Ra for the oxidizing gases. Ra and Rg represent the electrical resistance in air and after 10 min of gas exposure, respectively. The sensitivity was defined as the ratio of a sensor response change (ΔR) to a given gas concentration change (ΔC). The structures were tested, replicating the tests for each condition (analyte, concentration, and operating temperature) at least three times. Thus, the structures operated about 120 h for 4 weeks in total.

## 3. Results and Discussion

The aerosol-assisted chemical vapor deposited ZnO films were characterized by a greyish color that uniformly covered the substrates. The morphological, structural, and chemical characteristics of these films were consistent with those observed in our previous work on ZnO [17]. Specifically, SEM images of the films displayed quasi-aligned rods (NRs) with lengths of approximately 1.5 μm and an average diameter of 200 nm. Similarly, TEM showed crystalline structures with a marked planar spacing of 0.26 nm and rod-like morphology. Further analysis of the films using XRD corroborated the presence of diffraction patterns corresponding to a hexagonal ZnO phase (P63mc space group, ICCD card No. 00-005-0664), and XPS confirmed the presence of typical Zn 2p and O 1s spectra characteristic for the ZnO films, as shown in Figure S1, (Supplementary Materials). The films modified with Au, Ag, Fe_2_O_3_, and Cu_2_O in the second-step process showed morphological, crystalline, and chemical composition that corroborated the incorporation of these materials in the host ZnO structures, except for the Ag NPs modifications, which did not display clear evidence of Ag incorporation, neither by TEM nor by XPS, despite the apparent presence of Ag NPs at the ZnO surface after impregnation and annealing process (Figure S2, Supplementary Materials). Hence, hereafter, the results and discussion focus on the modification of ZnO with Au, Fe_2_O_3_, and Cu_2_O.

### 3.1. Gold-Modified Zinc Oxide Films

**Figure 1:**
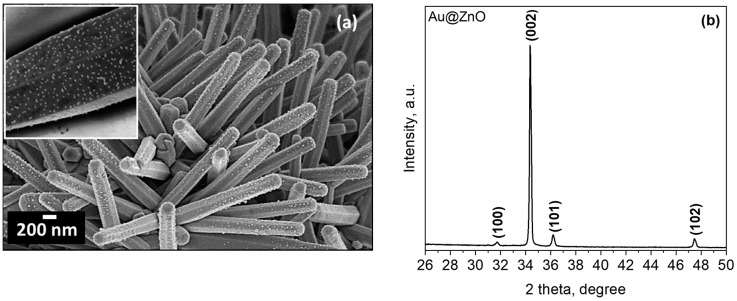
(**a**) SEM images (the inset shows a magnified image of a single Au decorated ZnO rod). (**b**) XRD diffraction pattern of the Au@ZnO structured film; all of the diffraction peaks in the data can be indexed to a hexagonal ZnO phase (P63mc space group, ICDD card No. 00-005-0664).

Figure 1a shows the morphology of the gold-modified zinc oxide films (Au@ZnO) recorded by SEM. The SEM images display the ZnO structured films with uniform rod-like morphology and the presence of equally distributed NPs due to the incorporation of Au NPs. XRD of the Au@ZnO films (Figure 1b) displays diffraction patterns connected with the hexagonal ZnO phase, in agreement with our previous observations [17], and an absence of Au diffraction peaks. This is probably connected with the high dispersion of the Au NPs over the film, their small size, and their low amount in the film.

**Figure 2:**
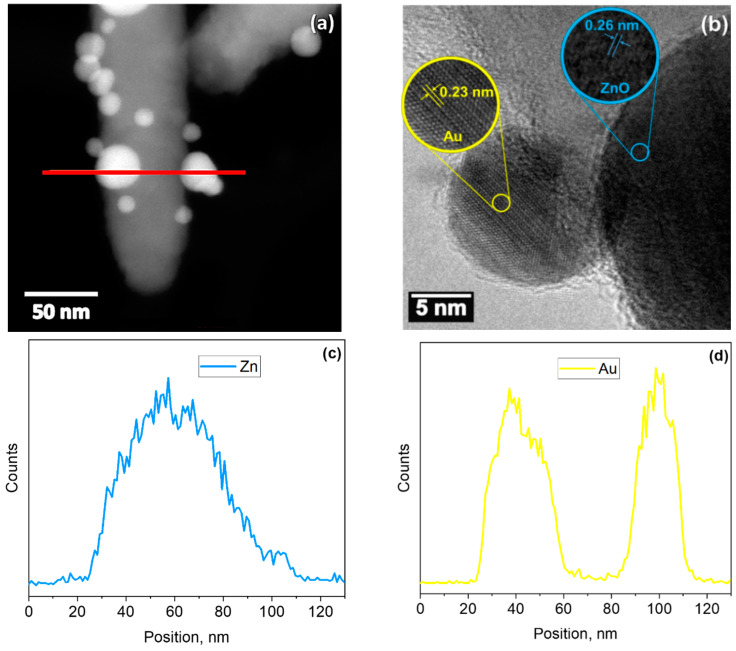
TEM image of an Au-modified ZnO rod. (**a**) Low-magnification STEM image, (**b**) high-resolution TEM image, and EDS line-scan profiles corresponding to the (**c**) ZnO rod and (**d**) Au nanoparticles recorded along the red line in (**a**).

The STEM image of the Au@ZnO structures in Figure 2a confirms the rod-like morphology of ZnO and its decoration with randomly located spherical NPs with diameters between 5 and 40 nm. HRTEM of these particles (Figure 2b) reveals the presence of highly ordered crystalline lattices with interplanar spacings of 0.26 nm and 0.23 nm, consistent with the hexagonal ZnO (002) [17] and cubic Au (111) planes (Fm-3m space group, ICDD card No. 00-004-0784), respectively. Similarly, the energy dispersive spectroscopy (EDS) line-scan profiles in Figure 2c,d show the components recorded on the particle along the red line (Figure 2a) and confirm the relative locations of the elements and the incorporation of Au NPs at the ZnO rod surface.

**Figure 3:**
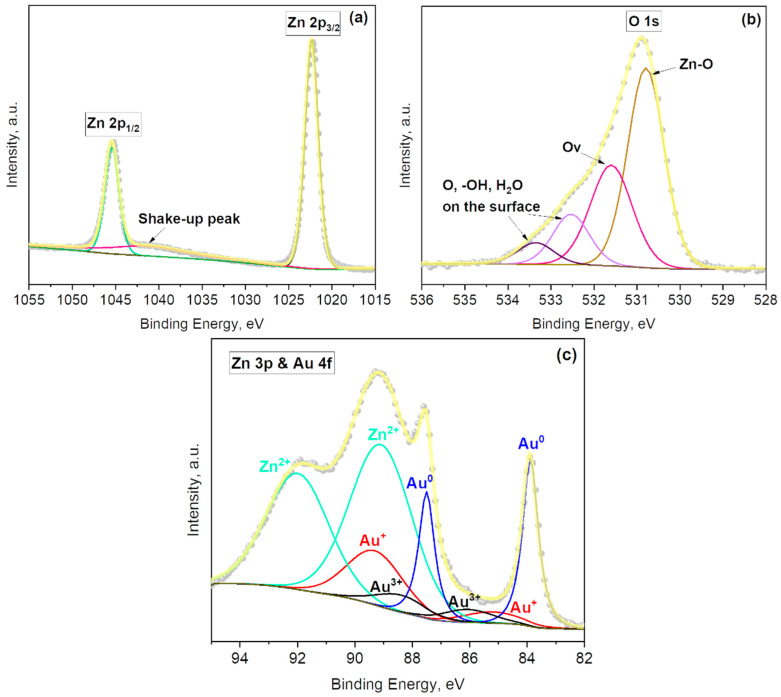
(**a**) Zn 2p, (**b**) O 1s, and (**c**) Au 4f and Zn 3p core-levels XPS spectra recorded on the Au@ZnO films. Grey dots represent the raw data, the solid yellow line corresponds to the envelope/fitting curve, and the other colored curves to the deconvoluted components.

XPS was used to analyze further the elemental and chemical composition of the Au-modified ZnO films. The main core-level spectra and their corresponding components are shown in Figure 3. The Zn 2p core-level spectrum recorded on the Au@ZnO film (Figure 3a) shows the typical Zn 2p doublet appearing at characteristic binding energies (1022.3 eV for Zn 2p_3/2_ and 1045.4 eV for Zn 2p_1/2_), together with a shake-up peak at 1040.7 eV. The binding energy separation (ΔBE = 23.1 eV) of this doublet is in agreement with the literature [30], and the ΔBE registered for the Zn 2p core-level peaks of the non-modified ZnO films (see Figure S1a, Supplementary Materials). However, it is noticed that the Zn 2p peaks of Au@ZnO are located on slightly higher binding energies (~0.7 eV) than those of ZnO, probably due to the incorporation of Au NPs into the ZnO film.

The deconvolution of the O 1s XPS spectrum of the Au@ZnO films indicates the presence of four peaks centered at 530.8 eV, 531.6 eV, 532.5 eV, and 533.4 eV (Figure 3b). The first component corresponds to the Zn–O bonds, the second to oxygen defects in the matrix of metal oxides related to oxygen vacancies, and the third and fourth to the physical or chemical adsorbed oxygen, hydroxides, and H_2_O on the surface of Au@ZnO film [31,32,33,34]. The number of components is similar to those found on the non-modified ZnO films, although their positions are minimally shifted to higher energy values (Figure S1b, Supplementary Materials).

Figure 3c displays the Au 4f spectrum of the Au@ZnO films. The deconvolution of this spectrum indicate the presence of six components assigned to different states of gold (Figure 3c), Au^0^ (at 83.9 eV and 87.5 eV), Au^+^ (at 85.2 eV and 89.3 eV), and Au^3+^ (at 86.1 eV and 88.4 eV) [35,36]. Moreover, two additional components are located at 89.1 eV and 92.0 eV, which originate from the Zn 3p emission band [37]. The formation of Au oxidized species in gold nanoparticles was reported previously and is plausible, particularly in small well-dispersed clusters [38]. An evaluation of the Au@ZnO films after the aging process at 310 °C in the same atmosphere used for the gas sensing tests (i.e., air and target gases, see Section 3.4) corroborated the stability of the gold nanoparticles incorporated at the ZnO surface and their chemical state. Quantification of the components related to gold indicate higher amounts of metallic gold (2.6 at.%) compared to Au^+^ (1.8 at.%) and Au^3+^ (0.7 at.%); this represents a 51%, 35%, and 14% of Au^0^, Au^+^, and Au^3+^, respectively, from the total gold content at the Au@ZnO sample.

### 3.2. Iron-Modified Zinc Oxide Films

**Figure 4:**
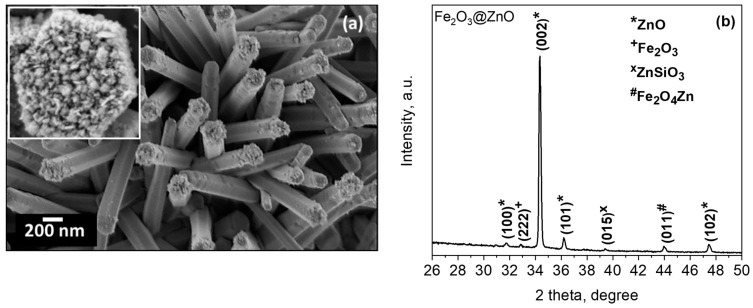
(**a**) SEM images (the inset shows a magnified image of the top of the Fe decorated ZnO rod) and (**b**) XRD diffraction pattern of the Fe_2_O_3_@ZnO structured film.

The SEM images of the iron-modified zinc oxide films (Fe_2_O_3_@ZnO) are presented in Figure 4a. These images show the modification of the ZnO structures with particles of flake-like morphology particularly concentrated on the top of the rods (inset in Figure 4a). The nanoflakes (NFs) are varied in size and do not exceed 20 nm in thick and 100 nm in diameter. The XRD pattern of these films corroborates the presence of the hexagonal ZnO phase determined earlier [17] and the appearance of new low-intensity diffractions. The first at 33.0° 2θ corresponds to the (222) plane of the cubic Fe_2_O_3_ (Ia−3 space group, ICSD Card No. 108905) and is consistent with previous Fe_2_O_3_ structures deposited by AACVD [39]. The second and third at 39.4° 2θ and 44.0° 2θ are connected with the rhombohedral ZnSiO_3_ (R-3 space group, ICSD Card No. 340575) and cubic Fe_2_O_4_Zn (Fd−3m space group, ICSD Card No. 91940), most likely present at the interfaces of Fe_2_O_3_/ZnO and ZnO/Si (from the substrate), respectively. This is consistent with our previous observation for similarly structured films [25].

**Figure 5:**
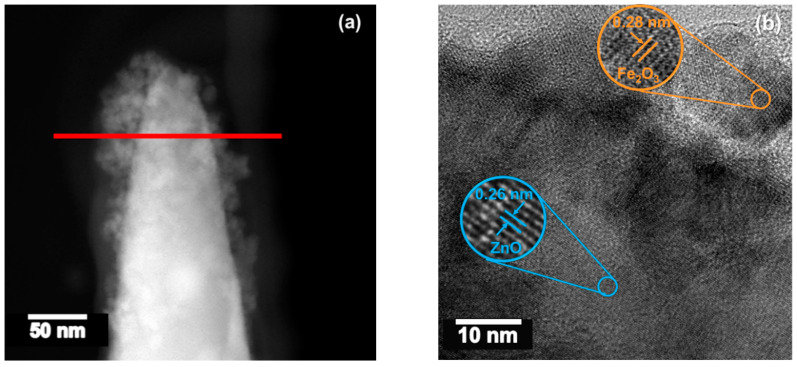

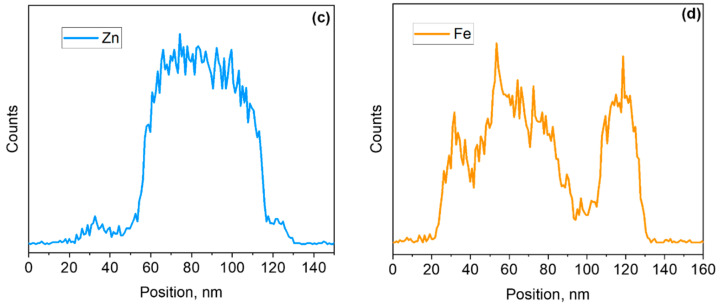
TEM image of a ZnO rod modified with Fe_2_O_3_. (**a**) Low-magnification STEM image, (**b**) high-resolution TEM image, and EDS line-scan profiles corresponding to (**c**) Zn and (**d**) Fe recorded along the red line in (**a**).

STEM images of the Fe_2_O_3_@ZnO structures (Figure 5a) showed a rod-like morphology covered by tiny particles. HRTEM images of these particles (Figure 5b) revealed crystalline structures with lattice spacings of 0.26 nm and 0.28 nm, assigned to the (002) plane of the hexagonal ZnO and the (222) plane of the cubic phase of the Fe_2_O_3_ identified by XRD, respectively. EDS line-scan profiles (Figure 5c,d) recorded along the red line in Figure 5a confirm the presence of Zn and Fe in the rod area and only Fe in the outer area of the rod.

**Figure 6:**
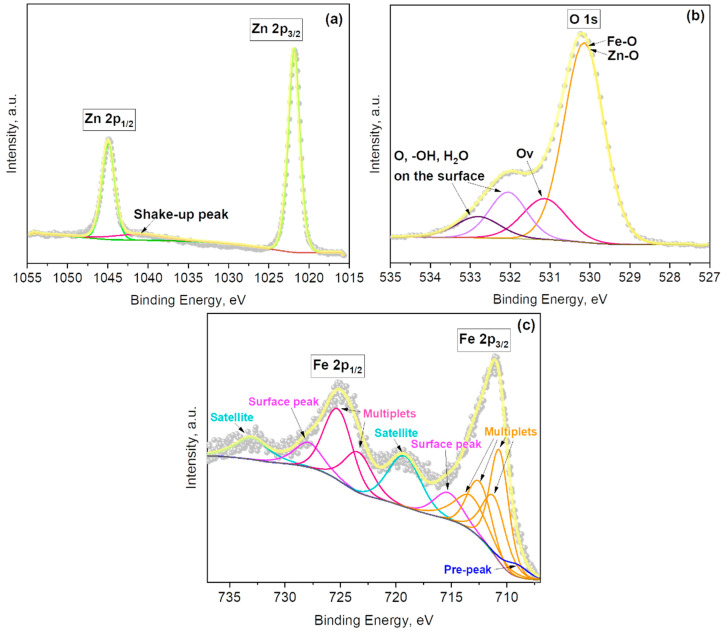
(**a**) Zn 2p, (**b**) O 1s, and (**c**) Fe 2p core levels XPS spectra recorded on the Fe_2_O_3_@ZnO film. The grey dots represent the raw data, the yellow solid line corresponds to the envelope/fitting curve, and the other colored curves to the deconvoluted components.

The surface chemical state of the Fe_2_O_3_@ZnO films characterized by XPS is presented in Figure 6. Similar to our previous observations on Au@ZnO films, the Zn 2p core-level spectrum of the Fe_2_O_3_@ZnO films showed a doublet (ΔBE = 23.2 eV) with the Zn 2p_3/2_ and Zn 2p_1/2_ peaks centered at 1021.8 eV and 1044.9 eV (Figure 6a), respectively, in agreement with the previous literature [25]. The Zn 2p core-level peaks from Fe_2_O_3_@ZnO show nearly identical positions to those of ZnO films and a slight shift to lower binding energies in relation to those of Au@ZnO films (see Figure S1a, Supplementary Materials, and Figure 3a).

The O 1s spectrum of the Fe_2_O_3_@ZnO films (Figure 6b) reveals the presence of four components. The first of them, with binding energy at 530.1 eV, corresponds to oxygen lattice bonds (Fe–O and Zn–O). The second component at 531.1 eV is assigned to the oxygen defects in the matrix of metal oxides due to oxygen vacancies. The third component, at 532.1 eV, and the fourth component, at 532.8 eV, are associated with the presence of physical or chemical adsorbed oxygen, hydroxides, and H_2_O on the surface of the structures, similarly to Au@ZnO film. These results indicate the Fe^3+^ ion incorporation into the ZnO lattice in agreement with the previous O 1s spectrum recorded on Fe_2_O_3_@ZnO films [25]. This is also consistent with the XRD results, which demonstrated the presence of the Fe_2_O_4_Zn compound at 44.0° 2θ (Figure 4b). Apart from the presence of one additional O 1s component in the Fe_2_O_3_@ZnO spectra compared to that of ZnO and Au@ZnO, one can also observe slight differences in the component positions, with the O 1s components of Fe_2_O_3_@ZnO showing higher binding energies (~0.4 eV) than the O 1s components of ZnO (Figure S1b, Supplementary Materials).

The Fe 2p spectrum displayed in Figure 6c shows two characteristic peaks at 711.1 eV and 725.0 eV, accompanied by two satellite peaks at 719.2 eV and 733.0 eV obtained from the photoelectrons emitted from Fe 2p_3/2_ and Fe 2p_1/2_, respectively. In total, the Fe 2p spectrum contains eleven components: four multiplets of Fe 2p_3/2_ peak, two multiplets of Fe 2p_1/2_ peak, two satellite peaks, two surface peaks, and one characteristic pre-peak usually present in the Fe 2p_3/2_ spectrum. The results and the binding energies of the peaks are in good agreement with the literature and suggest a Fe^3+^oxidation state for iron [25,40,41]. The total content of iron in the sample amounts is 7.5 at.%.

### 3.3. Copper-Modified Zinc Oxide Films

**Figure 7:**
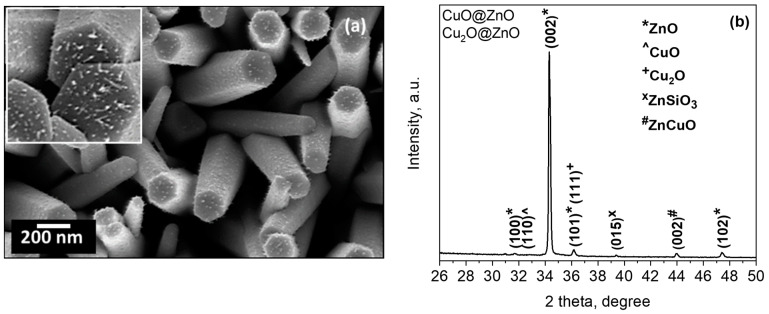
(**a**) SEM images (the inset shows a magnified image of the Cu decorated ZnO rods) and (**b**) XRD diffraction pattern of the copper-modified zinc oxide structured films.

SEM images of the copper-modified zinc oxide films also showed the presence of NPs distributed at the top and along the rod walls (Figure 7a). The XRD pattern of these films (Figure 7b) showed the intense diffractions that correspond to the hexagonal zinc oxide identified above with additional diffractions at 32.9° 2θ, 39.4° 2θ, and 44.0° 2θ. The first is connected to the (110) plane of the monoclinic copper(II) oxide phase (C12/c1 space group, ICSD Card No. 98-062-8618). The second and third are assigned to the ZnSiO_3_ (R-3 space group, ICSD Card No. 340575) and the cubic ZnCuO (0.85/0.15/1) compound (Fm-3m space group, ICSD Card No. 181023), both present most likely at the interface of ZnO with the modifier NPs and the Si substrate, respectively. The presence of diffraction peaks from the copper(I) oxide phase is not evident, probably due to the low amount of particles and their broad distribution. However, their presence is not ruled out as the (101) diffraction of ZnO overlaps the highest intensity diffraction peak of copper(I) oxide phase generally appearing at 36.4 2θ, e.g., for the ICSD Card No. 00-005-0667.

**Figure 8:**
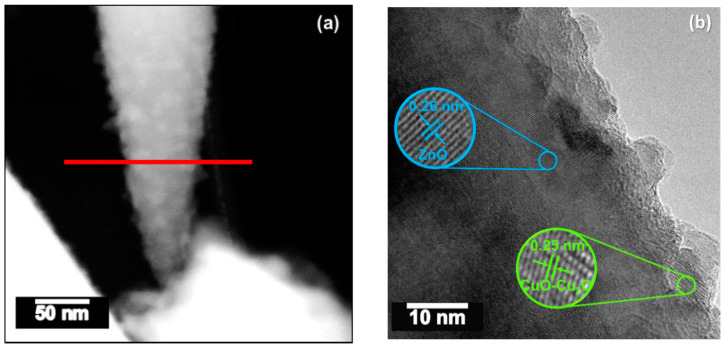

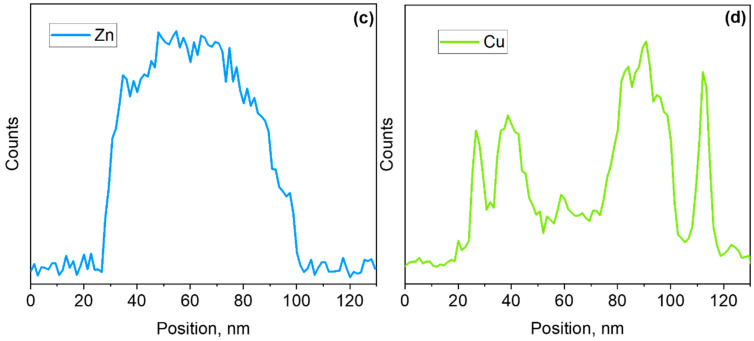
TEM images of a copper-modified ZnO rod. (**a**) Low-magnification TEM image, (**b**) high-resolution TEM image, and EDS line-scan profiles of the marked line in (**a**) corresponding to (**c**) Zn and (**d**) Cu.

The STEM and HRTEM images in Figure 8a,b show the crystalline copper-modified zinc oxide rods covered by spherical NPs. These particles display lattice spacings of 0.26 nm and 0.25 nm, first corresponding to the (002) plane of the hexagonal ZnO phase and second to either the (002) plane of the monoclinic copper(II) oxide or (111) cubic copper(I) oxide phase inferred by XRD. The EDS line-scan profiles of the components recorded along the red line in Figure 8a demonstrate the presence of Zn (Figure 8c) and Cu (Figure 8d) in the particles. These profiles also suggest that the CuO-Cu_2_O NPs are between 5 and 25 nm in size.

**Figure 9:**
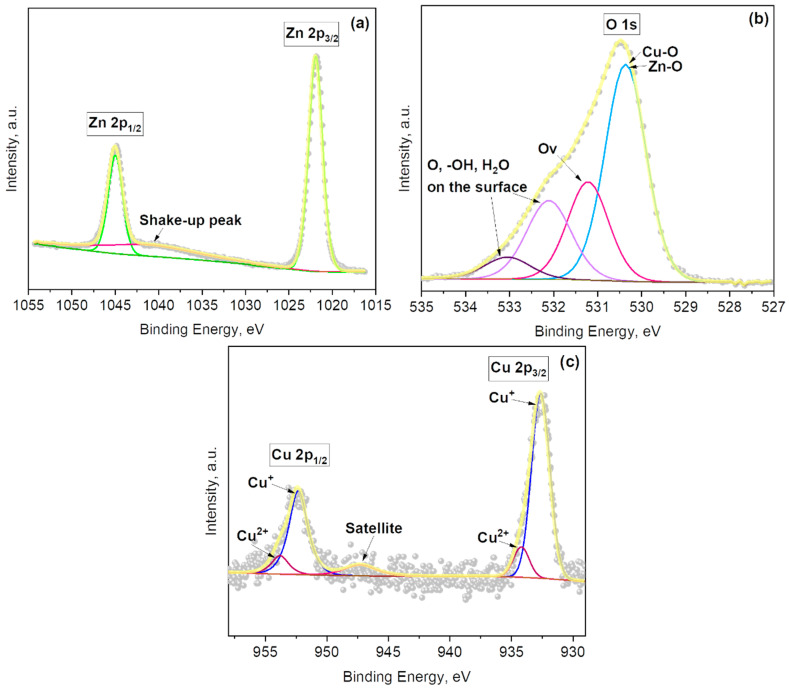
(**a**) Zn 2p, (**b**) O 1s, and (**c**) Cu 2p core levels XPS spectra for Cu_2_O@ZnO film. Grey dots represent the raw data, the solid yellow line corresponds to the envelope/fitting curve, and the other colored curves to the deconvoluted components.

An XPS analysis of the copper-modified zinc oxide films reported characteristic Zn 2p_3/2_ and Zn 2p_1/2_ core level peaks at 1021.9 eV and 1045 eV, respectively, separated by 23.1 eV and with a shake-up peak at 1040.3 eV (Figure 9a), similarly to previous studies [25]. These results are comparable to those for Fe_2_O_3_@ZnO as the Zn 2p doublet is located almost at the same binding energies for the copper-modified zinc oxide and Fe_2_O_3_@ZnO. Consequently, the Zn 2p doublet of the copper-modified zinc oxide films is located at higher and lower binding energies than those registered for the ZnO and Au@ZnO films, respectively (see Figure S1a, Supplementary Materials, and Figure 3a).

The O 1s spectrum (Figure 9b) of the copper-modified zinc oxide films contains four components. These are located at 530.4 eV (from Cu–O and Zn–O bonds), 531.2 eV (from oxygen defects in the matrix of metal oxides related to oxygen vacancies), and 532.1 eV and 533.0 eV (from chemisorbed oxygen, hydroxides, and H_2_O on the surface of the copper-modified zinc oxide film). These components are in agreement with the O1s spectra of the previously described materials.

The copper-modified zinc oxide films also displayed two doublets with components in Cu 2p_3/2_ at 932.6 eV and 934.2 eV, and Cu 2p_1/2_ at 952.3 eV and 953.9 eV (Figure 9c). The doublet with lower intensities (at 932.6 eV and 952.3 eV) indicates the presence of a Cu^+^ oxidation state from Cu_2_O, while the doublet with higher intensities (at 934.2 eV and 953.9 eV) corresponds to Cu^2+^ oxidation state from CuO [42]. The additional broad peak with low intensity in the area between 940 eV and 950 eV could be assigned to the satellite peak coming from the Cu^2+^ component [43]. This is consistent with the XRD and TEM analysis above, in which both species were also inferred. Quantification of the Cu^+^ and Cu^2+^ species at the surface of the samples suggest the presence of a higher amount of Cu_2_O (0.93 at.%) than CuO (0.13 at.%) at the surface of the samples; i.e., 87.7% of Cu_2_O and 12.3% of CuO from the total content of copper oxides at the copper-modified zinc oxide sample. Therefore, hereafter the discussion centers on the major copper oxide component (i.e., Cu_2_O), and the copper-modified zinc oxide films are abbreviated as Cu_2_O@ZnO.

### 3.4. Gas Sensing Tests

Gas sensing tests of the structured films (i.e., ZnO, Au@ZnO, Fe_2_O_3_@ZnO, and Cu_2_O@ZnO) were carried out to various gases (nitrogen dioxide, ethanol, acetone, carbon monoxide, and toluene) by using dc resistance measurements. To determine the thermal dependence of the response, the structures were initially tested to 80 ppm of ethanol at various operating temperatures, from 200 to 310 °C. These results showed that the dynamic and magnitude of the response improved as the operating temperature increased, registering the highest and fastest responses at the highest temperature tested. Therefore, the subsequent tests were carried out at 310 °C using four samples of each structure type and performing at least three replicates for each condition.

**Figure 10:**
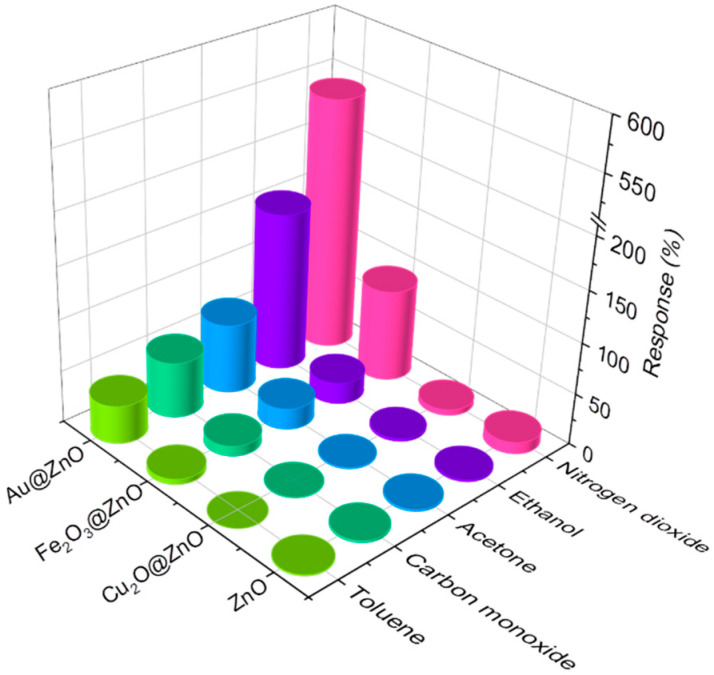
Responses towards 10 ppm of nitrogen dioxide and 80 ppm of ethanol, acetone, carbon monoxide, and toluene for the ZnO, Au@ZnO, Fe_2_O_3_@ZnO, and Cu_2_O@ZnO sensors.

Figure 10 summarizes the obtained results towards 10 ppm of nitrogen dioxide and 80 ppm of ethanol, acetone, carbon monoxide, and toluene. Overall, results reveal a significant improvement of the ZnO response due to its modification with Au NPs. One can also notice that the ZnO rods modified with Fe_2_O_3_ nanoflakes show an improvement compared to the non-modified ZnO, although not as high as that reported for the ZnO modified with Au. In contrast, the response observed for the ZnO structures modified with Cu_2_O NPs showed a decrement of the response compared to the non-modified ZnO structures.

**Figure 11:**
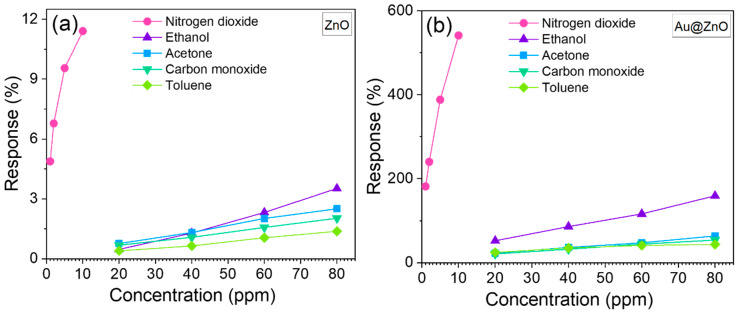

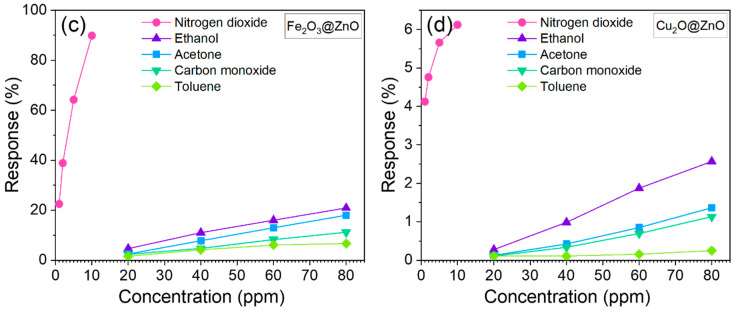
Response of the (**a**) ZnO (**b**) Au@ZnO, (**c**) Fe_2_O_3_@ZnO, and (**d**) Cu_2_O@ZnO sensors towards various concentrations of each target analyte.

Figure 11 displays the results obtained by testing the sensors to various concentrations of each gas (1 to 10 ppm for nitrogen dioxide and 20 to 80 ppm for ethanol, acetone, carbon monoxide, and toluene). Overall, the four structures demonstrate their highest responses to nitrogen dioxide, despite the lower concentrations of nitrogen dioxide compared to the other gases. The results also show a proportional increase in the response with the gas concentration. The tests corroborated the best response for the Au@ZnO structures among the other structures for all tested gases and applied concentrations. For instance, the Au@ZnO response to 10 ppm of nitrogen dioxide is about 47 times higher than that of ZnO (see Figure 11a,b). The difference in the response of these two structures to 80 ppm of ethanol is similar, showing around 45 times higher response for Au@ZnO compared to ZnO. For the low concentration, the difference in the responses of Au@ZnO and ZnO is still significant. For example, the responses of Au@ZnO sensors to 1 ppm of nitrogen dioxide and 20 ppm of ethanol are approximately 37 and 25 times higher, respectively, in comparison to the response of ZnO.

**Figure 12:**
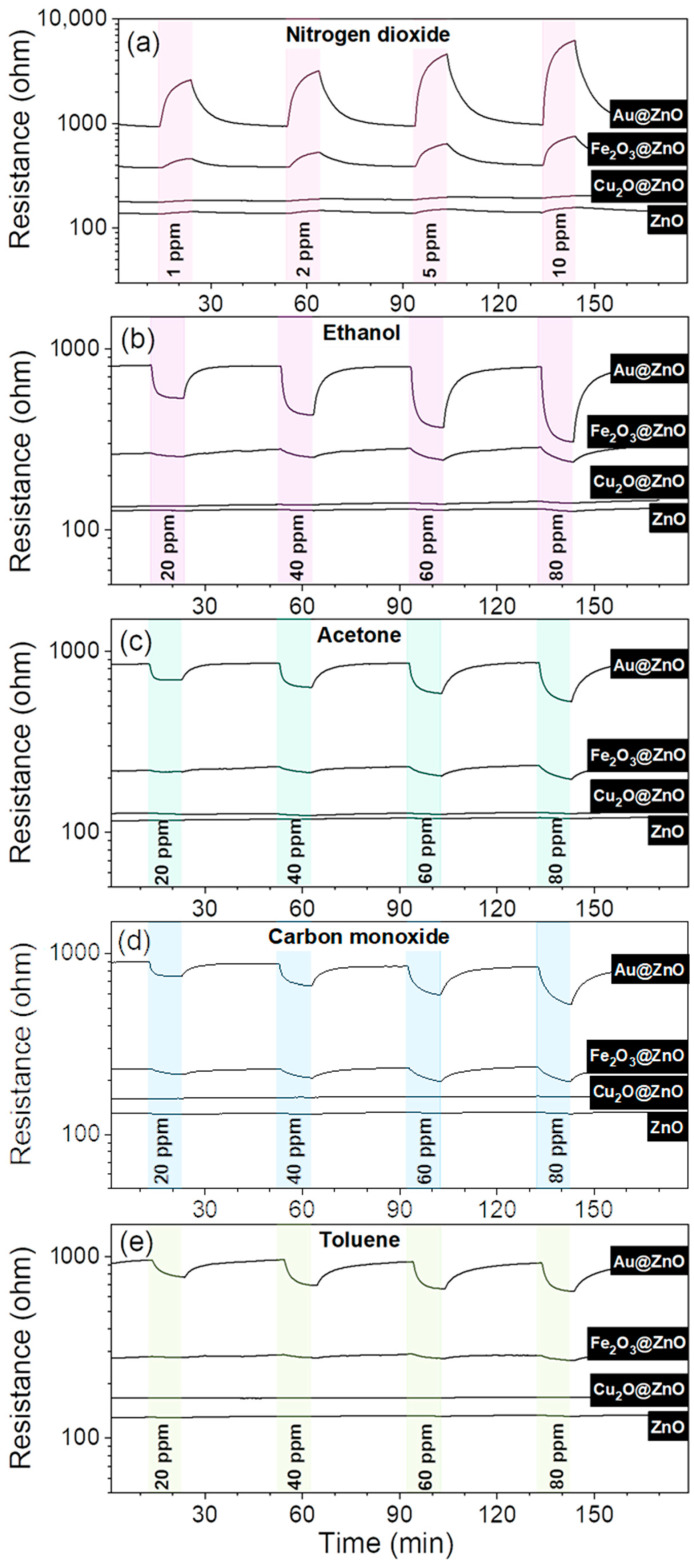
Electrical resistance changes to various concentrations of (**a**) nitrogen dioxide, (**b**) ethanol, (**c**) acetone, (**d**) carbon monoxide, and (**e**) toluene.

Figure 12 displays the electrical resistance changes recorded on the ZnO, Au@ZnO, Fe_2_O_3_@ZnO, and Cu_2_O@ZnO structures to each gas and concentration. Overall the structures indicate an n-type semiconducting behavior, reporting an increase in the electrical resistance to nitrogen dioxide (oxidizing gas) and a decrease in the resistance in front of reducing gases such as ethanol, acetone, carbon monoxide, and toluene. The responses are reversible for each analyte and concentration, showing a full recovery of the initial baseline resistance after purging the target analytes.

**Figure 13:**
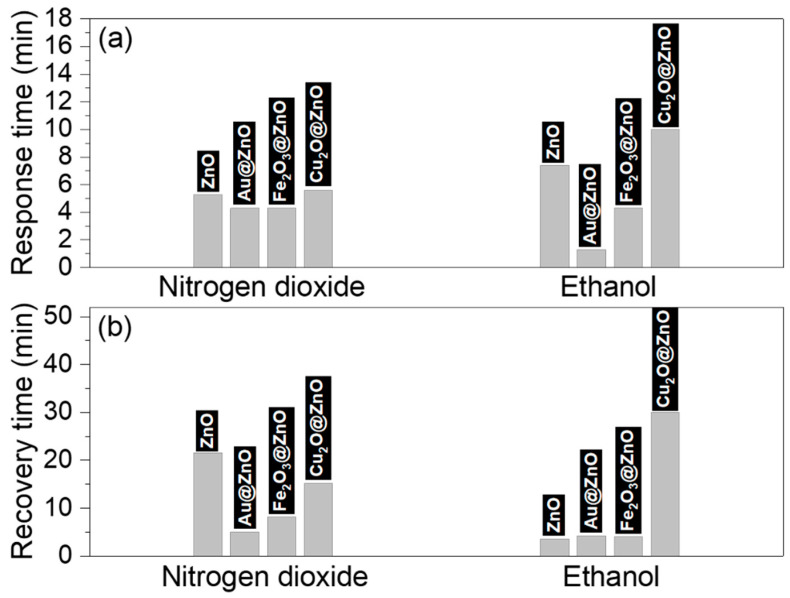
(**a**) Response and (**b**) recovery times to 1 ppm of nitrogen dioxide and 20 ppm of ethanol recorded with the non-modified and modified ZnO structures.

Figure 13 sums up the response (t_R_) and recovery (t_r_) time of each structure towards nitrogen dioxide and ethanol. As it can be noticed, the response time to nitrogen dioxide does not show significant changes among the four structures, showing periods between 4 and 6 min. However, this is different for the recovery time, which demonstrates faster recovery for the Au@ZnO (5 min) and Fe_2_O_3_@ZnO (8 min) structures compared to the ZnO (22 min) and Cu_2_O@ZnO (15 min) structures. The results for ethanol, in contrast, reveal the fastest response time for Au@ZnO (1.5 min) and nearly similar recovery time (4 min) for ZnO, Au@ZnO, and Fe_2_O_3_@ZnO.

A summary of the sensitivity of each structure to the target analytes in the tested concentrations range is presented in Table 1. These results evidence the high sensitivity of Au@ZnO and Fe_2_O_3_@ZnO structures to nitrogen dioxide with an estimated limit of detection (LOD) for each system of 26 and 114 ppb, respectively. The results also suggest an improvement of the selectivity of ZnO to nitrogen dioxide by its modification with Au or Fe NPs. The partial selectivity of Au@ZnO to ethanol among the other reducing gases is also worth it of mention.

The test of the sensors to reducing gases in humid ambient revealed sensor response losses by increasing the relative humidity (RH). In contrast, the test to nitrogen dioxide in humid ambient showed responses with a tendency to increase, although with poor stability and high signal noise. The greatest response changes in both cases were registered by increasing the humidity from 0% RH to 20% RH. Above this value, the changes were negligible due to the saturation of the response to relative humidity, as noticed previously for similar AACVD ZnO structures [44]. The loss of the response for ethanol, acetone, and carbon monoxide is displayed in Figure S3, Supplementary Materials. These results show that the Au@ZnO sensors lose 30% of their response to ethanol and acetone and 18% to carbon monoxide, whereas the loss of response for the Fe_2_O_3_@ZnO sensors is about 20% towards ethanol and acetone and 2% to carbon monoxide. The changes in the response due to humidity for the Cu_2_O@ZnO and ZnO sensors were minor, and the highest losses (8% and 2%) were recorded for Cu_2_O@ZnO to ethanol and ZnO to carbon monoxide.

### 3.5. Gas Sensing Mechanism

Chemoresistive gas sensors based on MOXs rely on the changes in the sensitive-material resistance upon exposure to target gases. These changes depend to a great extent on the gas analytes and their adsorption and desorption at the MOX surface. The gas sensing tests in this work demonstrate that the resistance changes of ZnO to nitrogen dioxide are significant compared to the other gases, and so that at first, we focus the discussion on the possible sensing mechanism of ZnO towards nitrogen dioxide.

**Figure 14:**
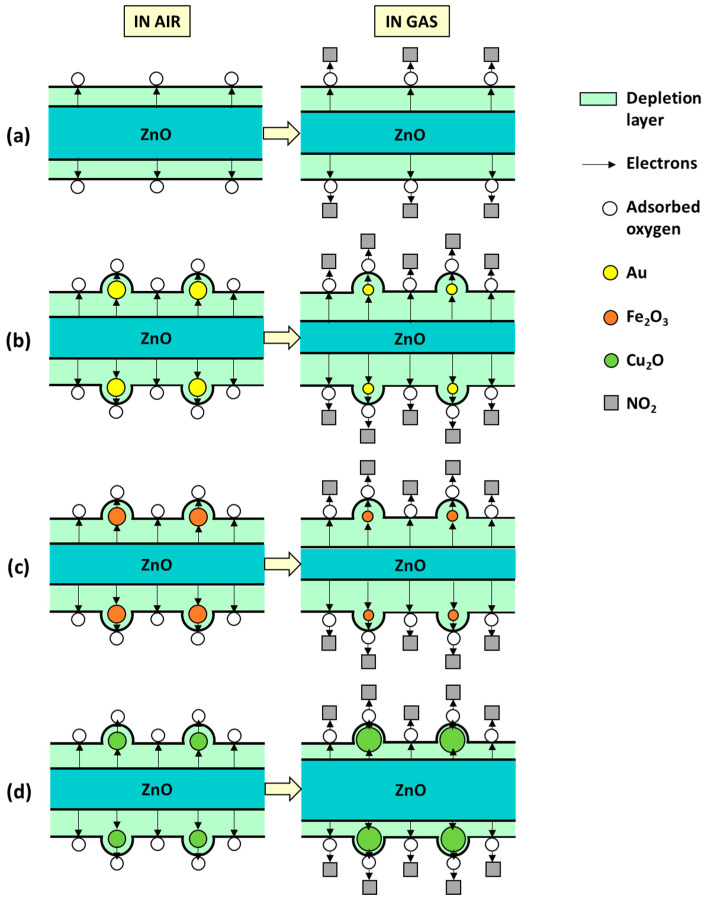
Schematic representation of the possible sensing mechanism of (**a**) ZnO, (**b**) Au@ZnO, (**c**) Fe_2_O_3_@ZnO, and (**d**) Cu_2_O@ZnO to nitrogen dioxide.

**Figure 15:**
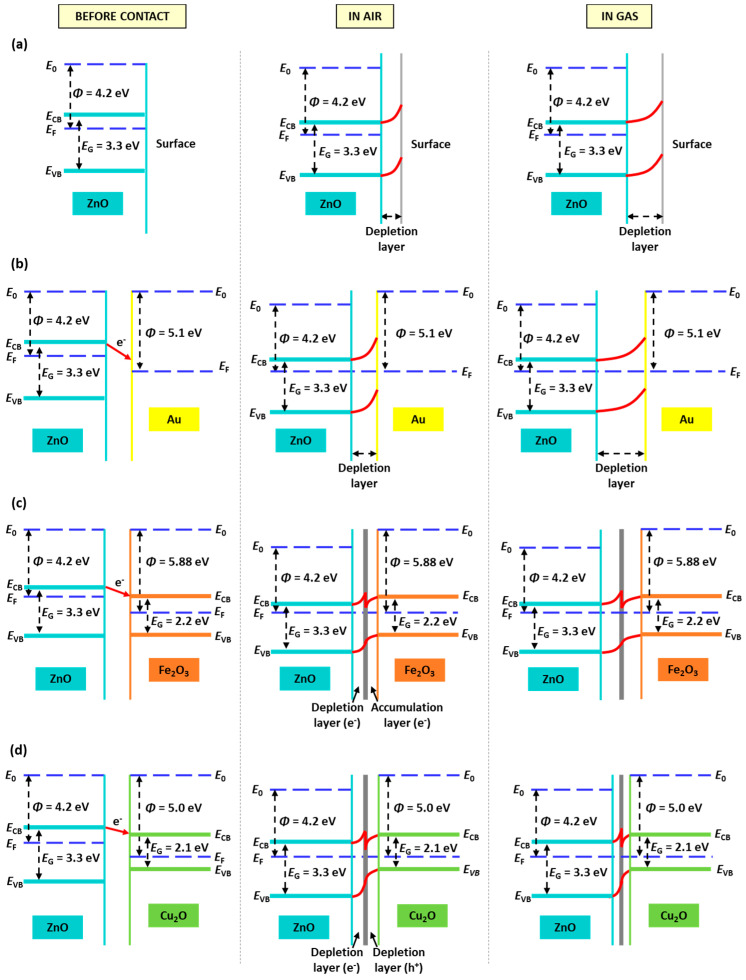
Energy band diagrams of (**a**) ZnO, (**b**) Au@ZnO, (**c**) Fe_2_O_3_@ZnO, and (**d**) Cu_2_O@ZnO films at room temperature and before contact between ZnO with Au, Fe_2_O_3_, or Cu_2_O (first column), after contact and in air (second column), and in gas (third column).

In the first phase, the sensing mechanism involves the interaction of the ZnO surface with air. Consequently, oxygen is adsorbed at the ZnO surface by trapping electrons from its conduction band and forming chemisorbed species in the form of O_2_^−^, O^−^, or O^2−^. However, due to the high operating temperatures employed in the test, O^−^ and O^2−^ species tend to be dominant at the surface [45]. This process leads to the formation of an electron depletion layer at the surface and, accordingly, an increase in the ZnO electrical resistance (Figure 14a and Figure 15a).

In the second phase, when the sensing material is exposed to a target gas, such as nitrogen dioxide, the adsorbed oxygen species react with the gas. As nitrogen dioxide is a strong oxidizing agent with electrophilic properties, it acts as an electron acceptor, pulling out electrons from the conduction band (CB). Consequently, the electron depletion layer width increases (Figure 14a and Figure 15a), leading to an increase in the sensor resistance. According to previous studies [46], the reaction between nitrogen dioxide and the formed oxygen species may be as follows:

(1) $${\mathrm{NO}}_{2}\left(\mathrm{gas}\right)+{\;O}_{2}^{-}+2e^{-}\to {\mathrm{NO}}_{2}^{-}\left(\mathrm{ads}\right)+2O^{-}\left(\mathrm{ads}\right)\;$$

The sensing mechanism of the Au, Fe_2_O_3_, and Cu_2_O modified ZnO structures, apart from the described above, involves additional synergistic effects due to the materials modifiers. These effects generally include spill-over of reactive species from the modifiers to ZnO and/or electronic effects due to the formation of extra nano-interfaces that facilitate the charge transfer during the gas–solid interactions at the surface. The modified materials can also have an impact on surface defects and oxygen vacancies (Ov) with respect to the pristine structure. This, in part, also influences the sensing performance of the material, to the extent that the oxygen vacancies act as donors modifying the baseline conductivity of the sensor material and consequentially the sensor response [47,48]. The XPS components associated with the oxygen vacancies in the results above indicate a higher amount of vacancies at the Au@ZnO films (19.1 at.%) compared to Cu_2_O@ZnO (15.4 at.%), Fe_2_O_3_@ZnO (10.6 at.%), and pure ZnO (7.8 at.%) films, which partially explains the better gas sensing performances of the modified Au@ZnO and Fe_2_O_3_@ZnO. However, this is not consistent with the Cu_2_O@ZnO results, which show a different behavior most likely influenced by the type of interface formed between these materials. The band energy of each system also has a role in the sensing response, and therefore below, we focus the discussion on the effect of each modifier material on the band bending and band alignment at the surface and the sensing properties of each ZnO-based system.

#### 3.5.1. Gold-Modified Zinc Oxide Films

The results above showed that the decoration of the pristine ZnO structures with Au improves the gas sensing performance of the first, similarly to the previous literature results for noble metal modified ZnO systems [24,49]. The chemical composition of the Au@ZnO films corroborated the incorporation of gold (Au^0^, Au^+^, Au^3+^) at the ZnO surface with a major presence of Au^0^ (2.6 at.%) than Au^+^ (1.8 at.%) or Au^3+^ (0.7 at.%), suggesting the sensing mechanism of Au@ZnO films rely mainly on the metallic Au state. Hence, the performance improvements could be linked particularly to two phenomena—the formation of nano Schottky junctions between Au and ZnO and the catalytic activity of Au NPs. However, the electronic influence of the Au oxidized species is not ruled out, which may enhance the interaction of the gold nanoparticles with ZnO and contribute to better gas sensing performance, as observed previously [26].

Schottky junctions are formed when contacting a noble metal (e.g., Au) and a MOX (e.g., ZnO) with different work functions (WF), 4.2 eV (ZnO) [50] and 5.1 eV (Au) [51], and consequentially different Fermi level energies. Since the WF of Au is higher than that of ZnO, most of the energy required to withdraw an electron from bulk to surface is higher compared to ZnO. Because of that, free electrons from ZnO are more prone to move from ZnO to Au until the Fermi energies become equal (Figure 15b). Therefore, in the first phase of the gas sensing mechanism, when Au@ZnO is exposed to air, the electron transferring from ZnO to Au leads to the adsorption of more oxygen species and, in turn, the formation of a thicker depletion layer compared to the pristine ZnO films for the same conditions. Consequently, the resistance of the Au@ZnO sensors is higher than that of the ZnO sensors. This is consistent with the electrical resistance registered for these sensors (Figure 12).

In the second phase of the mechanism, when Au@ZnO is exposed to nitrogen dioxide, adsorbed oxygen species will react with the gas, and more electrons will be consumed from the conduction band of the modified ZnO films compared to the non-modified one. This leads to a proportional increase in the electron depletion layer width (Figure 14b and Figure 15b) and, eventually, a higher increase in the Au@ZnO resistance compared to the ZnO sensors.

Additionally, the catalytic activity of Au NPs, as reported previously in the literature [52,53], may play a role in accelerating the kinetics of the surface reactions during the sensing mechanism. This is consistent with the better dynamics of the Au@ZnO sensor’s response (Figure 12), which shows faster response and recovery times, particularly for the reducing gases (Figure 13) in contrast to the ZnO sensors.

#### 3.5.2. Iron- and Copper-Modified Zinc Oxide Films

Although Fe_2_O_3_@ZnO and Cu_2_O@ZnO rely on similar gas sensing mechanism principles because both systems present the formation of nano-heterojunctions at the interface of the ZnO rods and the decorating MOX (Fe_2_O_3_ or Cu_2_O) particles, the difference between these materials lies in their type of conductivity. Cu_2_O reports p-type semiconductor behavior [54], whereas Fe_2_O_3_ may present either n- or p-type behavior [7]. Our previous studies, however, demonstrated that the Fe_2_O_3_ films obtained via the AACVD of FeCl_3_·6H_2_O have an n-type behavior with a typical increase in conductivity when exposed to reducing gases [39]. Therefore, hereafter we discuss the sensing mechanism of Fe_2_O_3_@ZnO and Cu_2_O@ZnO, considering their n- and p-type behavior, respectively.

Due to the different work function (5.88 eV [55]) and band-gap (2.2 eV [56]) of Fe_2_O_3_ compared to ZnO (4.2 eV and 3.3 eV [57], respectively), and higher position of the CB of ZnO, free electrons from the CB of ZnO migrate to the CB of Fe_2_O_3_. This is the opposite for the holes, which migrate from the valence band (VB) of Fe_2_O_3_ to that of ZnO until the Fermi levels of both materials reach equilibrium (Figure 15c). These processes increase the number of electrons near the surface of the system so that when Fe_2_O_3_@ZnO sensors are exposed to air, more oxygen species can be adsorbed at the surface in comparison to pristine ZnO as displayed in Figure 14c. Consequentially, in the gas stage (e.g., nitrogen dioxide), more gas molecules react with the adsorbed oxygen, and a higher amount of electrons is trapped, enhancing the response of the Fe_2_O_3_@ZnO sensors compared to Zn.

Similarly, Cu_2_O, with a work function of 5.0 eV and a band-gap of 2.1 eV [54], has a CB below that of ZnO, meaning that the transfer of electrons occurs from the ZnO side towards the Cu_2_O side as shown in Figure 15d. Analogously to the Fe_2_O_3_@ZnO sensor, more electrons will provide more adsorbed oxygen on the surface and more active sites for gas molecules to react (Figure 14d). However, in the case of p-type semiconductors, such as Cu_2_O, which have as main carriers holes, the ionosorption of oxygen favors the conduction by holes (Figure 15d), contrary to n-type semiconductors. Therefore, when the oxidizing gas NO_2_ comes in contact with a p-type semiconductor, NO_2_ will trap the electrons, which will increase further the numbers of holes at the interface [58] and decrease the resistance of the whole Cu_2_O@ZnO system partially; a similar effect was reported previously for instance for ZnO decorated with p-type Cr_2_O_3_ [59]. This is most likely the rationale behind the loss of sensitivity to nitrogen dioxide for the Cu_2_O@ZnO sensors compared to the pristine ZnO and the modified ZnO with gold and iron (see Table 1).

The presented mechanism described the sensing behavior of the different ZnO systems towards oxidizing gases (e.g., nitrogen dioxide). Reducing gases, such as ethanol, behave in an opposite way, providing electrons to ZnO and thus decreasing its resistance, as can be noticed in the electrical resistance changes in Figure 12b–e.

As we can conclude from the previous, all three systems are influenced by the incorporation of nano-interfaces, which provide slightly different gas sensing mechanisms that favor especially the sensing performance of the structures modified with Au due to the formation of Schottky junction and catalytic properties of Au. The formation of heterojunctions also shows advantages in the sensing performance of the structures modified with Fe, although it shows an opposite effect for the structures modified with Cu, most likely due to the difference in the conductivity type of Fe_2_O_3_ (n-type) and Cu_2_O (p-type) semiconductors. Generally, Fe_2_O_3_ and Cu_2_O could also act as catalyzers, but in light of the dynamic of response observed, their catalytic activity is not the main parameter that influences the response of these modified structures.

## 4. Conclusions

Zinc oxide structures were deposited and modified with Au, Fe_2_O_3_, and Cu_2_O nanoparticles. The results showed the formation of ZnO rods (approximately 1.5 μm long with an average diameter of 200 nm) as well as the incorporation of crystalline Au nanoparticles (diameters between 5 and 40 nm), Fe_2_O_3_ nanoflakes (thickness smaller than 20 nm and diameter of 100 nm), and Cu_2_O nanoparticles (diameters between 5 and 25 nm). Chemical composition determination by XPS revealed the presence of Fe in the form of oxide—Fe_2_O_3_; Cu in the form of two oxides—CuO and Cu_2_O, with the major presence of Cu_2_O; and Au in three oxidation states—Au^3+^, Au^+^, and Au^0^, with the content of metallic Au (2.6 at.%) being the highest among the other states. Gas sensing results showed the best response for the Au@ZnO sensors among the others, especially for nitrogen dioxide, with about 47 times higher response to 10 ppm of nitrogen dioxide compared to the ZnO sensors. The sensitivity (39.96% ppm^−1^) and limit of detection (26 ppb) reported for this modified material to nitrogen dioxide were also higher compared to the other sensor structures. This was attributed to a combination of factors, including the oxygen vacancies, the gas sensing mechanism influenced by the nano-interfaces formed between ZnO and Au, and possible catalytic effects of the Au nanoparticles.

## Figures and Tables

**Table 1:** Sensitivity (ΔR/ΔC) in % ppm^−1^ to ethanol, acetone, carbon monoxide, and toluene for concentrations between 20 and 80 ppm and nitrogen dioxide for concentrations between 1 and 10 ppm.

Analyte	ZnO	Au@ZnO	Fe_2_O_3_@ZnO	Cu_2_O@ZnO
Nitrogen dioxide	0.73	39.96	7.48	0.22
Ethanol	0.05	1.79	0.27	0.04
Acetone	0.03	0.68	0.26	0.02
Carbon Monoxide	0.02	0.55	0.15	0.02
Toluene	0.02	0.31	0.08	0.002

## Data Availability

Not applicable.

## Supplementary Materials

The following are available online at https://www.mdpi.com/article/10.3390/s21134509/s1. Figure S1: (a) Zn 2p, and (b) O 1s core levels XPS spectra for non-modified ZnO film. Grey dots represent the raw data, the solid yellow line corresponds to the envelope/fitting curve, and the other colored curves to the deconvoluted components. Figure S2: SEM images (the inset shows a magnified image of a single Ag decorated ZnO rod) of the Ag@ZnO structured film. Figure S3: Responses towards 80 ppm of ethanol, acetone, carbon monoxide for the ZnO, Au@ZnO, Fe_2_O_3_@ZnO, and Cu_2_O@ZnO sensors at 0% and 20% RH.
