# Supplementary material for: ZnO Structures with Surface Nanoscale Interfaces Formed by Au, Fe2O3, or Cu2O Modifier Nanoparticles: Characterization and Gas Sensing Properties
Source: Sensors (Basel). 2021 Jun 30;21(13):4509. doi: 10.3390/s21134509 (PMC8271897; doi:10.3390/s21134509)
Supplement: Supplementary file 1 [file sensors-21-04509-s001.zip › sensors-1273217-supplementary.pdf]

Supplementary Materials

ZnO structures with surface nanoscale interfaces formed by Au, Fe_2O_3, or Cu_2O modifier nanoparticles: characterization and gas sensing properties

Milena Tomić [1,2], Martha Claros [3], Isabel Gràcia [1], Eduard Figueras [1], Carles Cané [1], and Stella Vallejos [1,3*]

[1] Institute of Microelectronics of Barcelona (IMB-CNM, CSIC), Campus UAB, 08193 Cerdanyola del Vallès, Barcelona, Spain; milena.tomic@imb-cnm.csic.es (M.T.); isabel.gracia@imb-cnm.csic.es (I.G.); eduard.figueras@imb-cnm.csic.es (E.F.); carles.cane@imb-cnm.csic.es (C.C.)
[2] Autonomous University of Barcelona (UAB), Department of Electronic Engineering, Campus UAB, 08193 Cerdanyola del Vallès, Barcelona, Spain
[3] CEITEC - Central European Institute of Technology, Brno University of Technology, 61200 Brno, Czech Republic; martha.claros@ceitec.vutbr.cz (M.C.)
* Correspondence: stella.vallejos@imb-cnm.csic.es (S.V.); Tel.: +34 935 947700

S1. Results – Material characterization

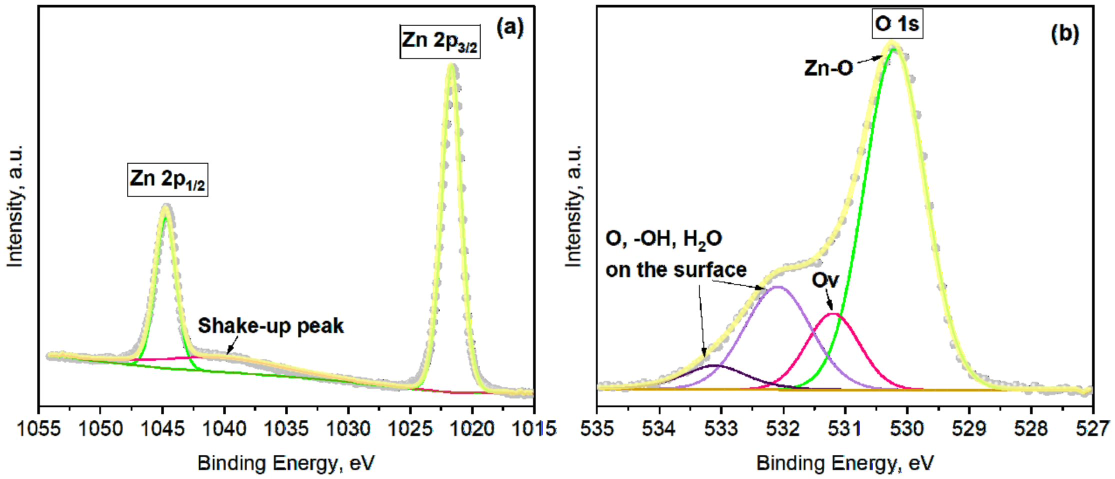

Figure S1. (a) Zn 2p, and **(b)** O 1s core levels XPS spectra for non-modified ZnO film. Grey dots represent the raw data, the solid yellow line corresponds to the envelope/fitting curve, and the other colored curves to the deconvoluted components.

The Zn 2p core-level spectrum recorded on non-modified ZnO film showed good agreement with the literature [1], proving the presence of Zn^{2+} state of zinc, with two main peaks at 1021.6 eV and 1044.7 eV that corresponds to the Zn $2p_{3/2}$ and Zn $2p_{1/2}$ peaks (**Figure S1a**), respectively, with the binding energy separation of 23.1 eV. Additional peak located at 1040.2 eV is related to the shake-up peak.

The O 1s XPS spectrum (**Figure S1b**) presents an asymmetric curve as reported earlier in the literature [2]. After the deconvolution, four different components are determined that indicate the presence of different oxygen species. The component centered at 530.2 eV is attributed to O^{2-} ions in the Zn-O bonding. The component at 531.2 eV is associated to O^- and O_2^- ions in the oxygen-deficient regions – oxygen vacancies. The components at 532.1 eV and 533.1 are connected to the presence of weak bonds from the physical or chemical adsorbed oxygen, hydroxides, and H_2O on the surface of ZnO film [3] [4] [5] [6].

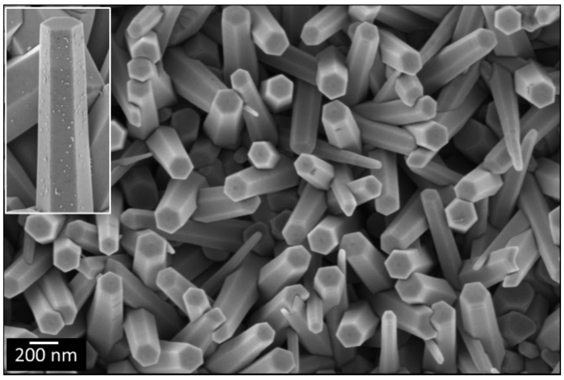

Figure S2. SEM images of the Ag@ZnO structured film (the inset shows a magnified image of a single Ag-modified ZnO rod).

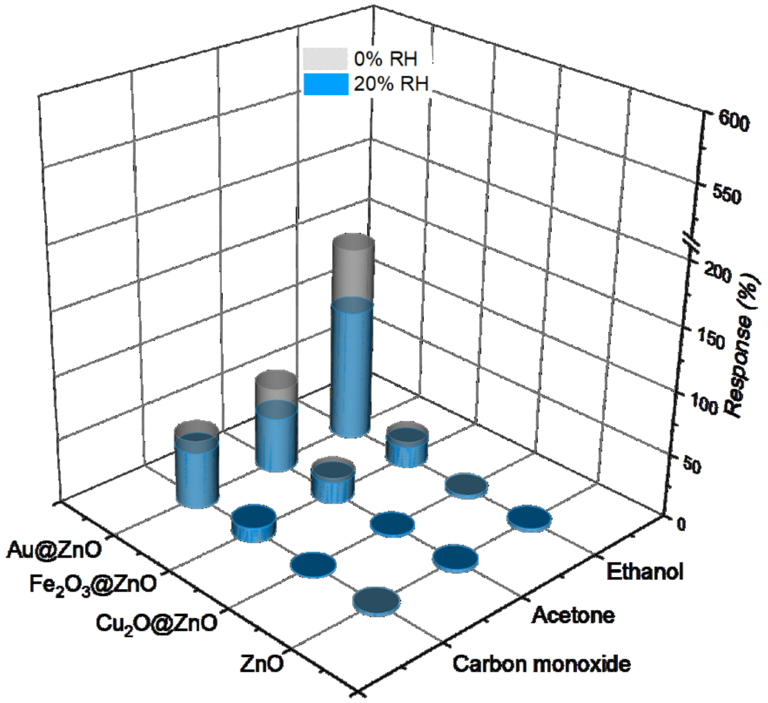

Figure S3. Responses towards 80 ppm of ethanol, acetone, carbon monoxide for the ZnO, Au@ZnO, Fe_2O_3@ZnO, and Cu_2O@ZnO sensors at 0% and 20% RH.

References:

[1] S. Vallejos *et al.*, "ZnO rods with exposed {100} facets grown via a self-catalyzed vapor-solid mechanism and their photocatalytic and gas sensing properties," *ACS Appl. Mater. Interfaces*, vol. 8, no. 48, pp. 33335–33342, 2016, doi: 10.1021/acsami.6b12992.

[2] J. H. Zheng, Q. Jiang, and J. S. Lian, "Synthesis and optical properties of flower-like ZnO nanorods by thermal evaporation method," *Appl. Surf. Sci.*, vol. 257, no. 11, pp. 5083–5087, 2011, doi: 10.1016/j.apsusc.2011.01.025.

[3] S. Jain, J. Shah, N. Negi, C. Sharma, and R. K. Kotnala, "Significance of interface barrier at electrode of hematite hydroelectric cell for generating ecopower by water splitting," *Int. J. Energy Res.*, vol. 43, pp. 4743–4755, 2019, doi: 10.1002/er.4613.

[4] H. Wang, S. Baek, J. Song, J. Lee, and S. Lim, "Microstructural and optical characteristics of solution-grown Ga-doped ZnO nanorod arrays," *Nanotechnology*, vol. 19, no. 7, p. 075607, Jan. 2008, doi: 10.1088/0957-4484/19/7/075607.

[5] E. De La Rosa *et al.*, "Controlling the growth and luminescence properties of well-faceted ZnO nanorods," *J. Phys. Chem. C*, vol. 111, no. 24, pp. 8489–8495, Jun. 2007, doi: 10.1021/jp071846t.

[6] L. J. Meng, C. P. Moreira de Sá, and M. P. dos Santos, "Study of the structural properties of ZnO thin films by x-ray photoelectron spectroscopy," *Appl. Surf. Sci.*, vol. 78, no. 1, pp. 57–61, May 1994, doi: 10.1016/0169-4332(94)90031-0.
